# 3D printing and thoracoscopy assisted MIPO in treatment of long-range comminuted rib fractures, a case report

**DOI:** 10.1186/s13019-019-0892-0

**Published:** 2019-04-29

**Authors:** Xuetao Zhou, Dongsheng Zhang, Zexin Xie, Menghui Chen, Yang Yang, Zheng Liang, Guoliang Zhang

**Affiliations:** Department of Cardiothoracic Surgery, The Third Hospital of Shijiazhuang City, Shijiazhuang, 050000 China

**Keywords:** 3D printing, Minimally invasive plate Oseoynthesis (MIPO), Multiple rib fractures, Thoracoscope

## Abstract

**Background:**

To investigate the application of 3D printing technology combined with percutaneous Minimally Invasive Plate Oseoynthesis (MIPO) and thoracoscopic techniques in the treatment of long comminuted rib fractures.

**Case presentation:**

One case of multiple rib fractures with abnormal respiratory disease (including rib 3 and 4 of long comminuted fractures) due to a fall injury was selected. The 3D model of comminuted rib fracture was reconstructed and printed according to the thin-layer CT scan results. After the fracture model was restored to the normal rib anatomy, the metal plate was accurately shaped according to the 3D rib shape.

**Conclusions:**

3D printing technology combined with MIPO technology under thoracoscopy in the minimally invasive treatment of long-range comminuted rib fractures, greatly reduced the time and improved the accuracy of intraoperative fixation, reduced the difficulty of surgery, patient injury, and perfectly reconstructed the chest wall. Application of the 3D printing technique to make the rib model and pre-mold the metal plate combined the thoracoscopic MIPO technology provides less invasive and accurate individualized treatment for complex rib fractures.

## Background

The traditional treatment of rib fractures is mainly conservative treatment [1].In recent years, with the continuous availability of new rib internal fixation materials and equipment, the development of surgical treatment has been greatly promoted, and the operation has also been simplified and minimally invasive [2]. The indications for internal fixation of rib fractures have also been constantly updated and broadened [3], but high rib fractures, especially behind the scapula, are complex and challenging issues for the surgeon. Traditional operation methods are not only difficult to operate, but also cause great damage to patients. However; the MIPO technique does not break the skin of the fracture end, and fix the fracture through the periosteal subcutaneous tunnel without destroying the blood supply of periosteum. With the rapid development of minimally invasive techniques in various medical fields, the use of orthopaedic MIPO technology and thoracoscopic technology, combined with 3D printing technology to rebuild the 3D model, preoperatively shaping the metal bone plate brings great convenience to surgery and greatly reduces patient injury.

## Case presentation

### Typical case

**Fig. 1:**
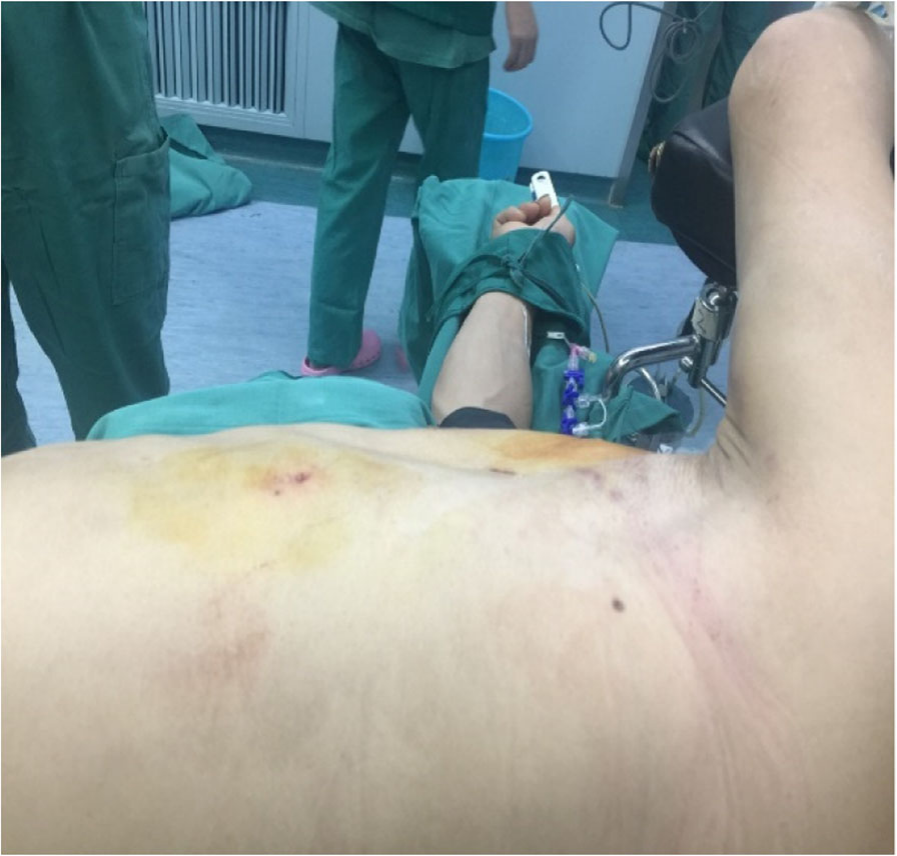
Observation of the patient and his rib fractures. Patient’s anterior chest wall depression.

The patient was a 61-year-old man with multiple left rib fractures (1–6 ribs), left pneumothorax, left lung contusion, and left thoracic subcutaneous emphysema due to a fall injury. The examination showed a partial depression in the left front rib and abnormal breathing (see Fig. 1).

**Fig. 2:**
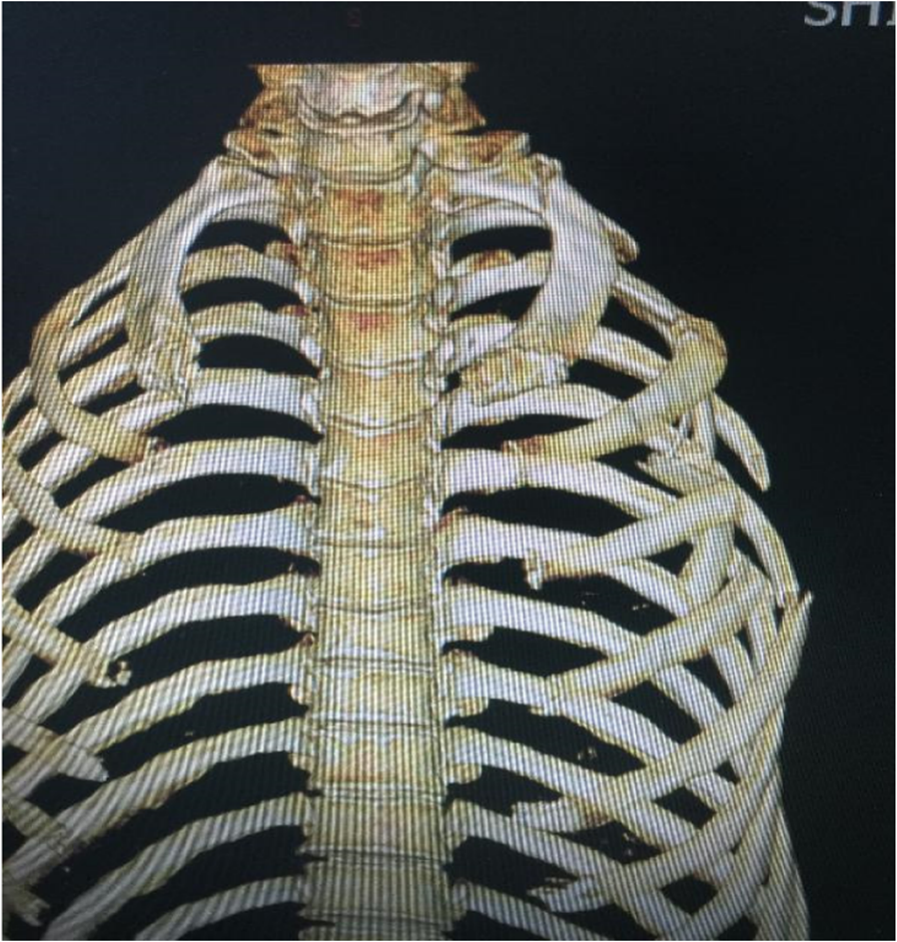
Multiple rib fractures on the left side, including comminuted fractures of 3 and 4 ribs

Admission chest CT examination: 1–6 rib fractures on the left side (of which 3, 4 ribs are long comminuted fractures (see Fig. 2)); left pneumothorax, left traumatic wet lung; a small amount of liquid pneumothorax on the left side.

Patient was given early chest straps, multiparametric monitoring, analgesia, and oxygen therapy. The chest pain was still severe. The visual analogue scale scored 7–8 points for the pain at rest and 9 points for the cough.

### Surgical methods

**Fig. 3:**
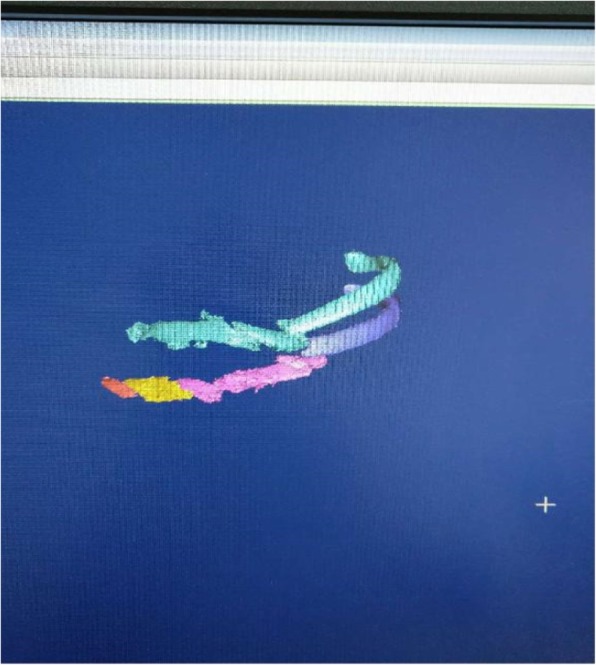
Rib model Reconstruction. Reconstruction of the 3D model from CT thin-layer scan results

**Fig. 4:**
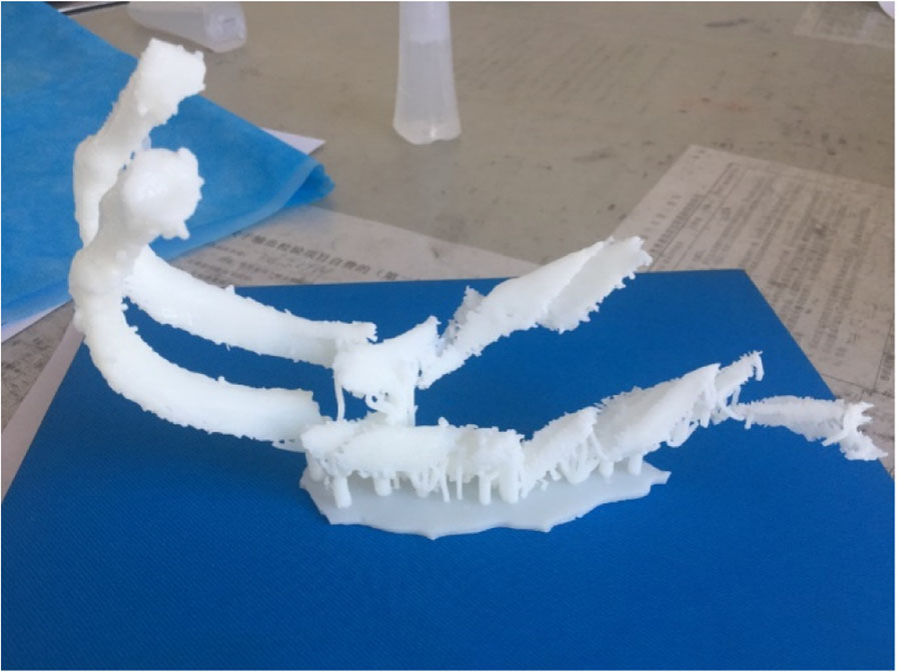
Preoperative Reconstruction of the 3D model from CT thin-layer scan results prepared by 3D printing technique. Preoperative 3D reconstruction of the 3rd and 4th rib images; 3, 4 rib model prepared by 3D printing technique.

Physical examination revealed that the left chest wall was recessed and abnormally breathed. The CT scan of the rib showed a long comminuted fracture of 3 and 4 ribs. The key to successful operation was the reduction and fixation of these two rib fractures. A preoperative CT scan was performed to reconstruct the 3D model based on the scan results (see Fig. 3), and 3D printing technology was used to prepare 3 and 4 rib models (see Fig. 4). The three D print models of each fracture segment of the two ribs were adherently reconstructed.

**Fig. 5:**
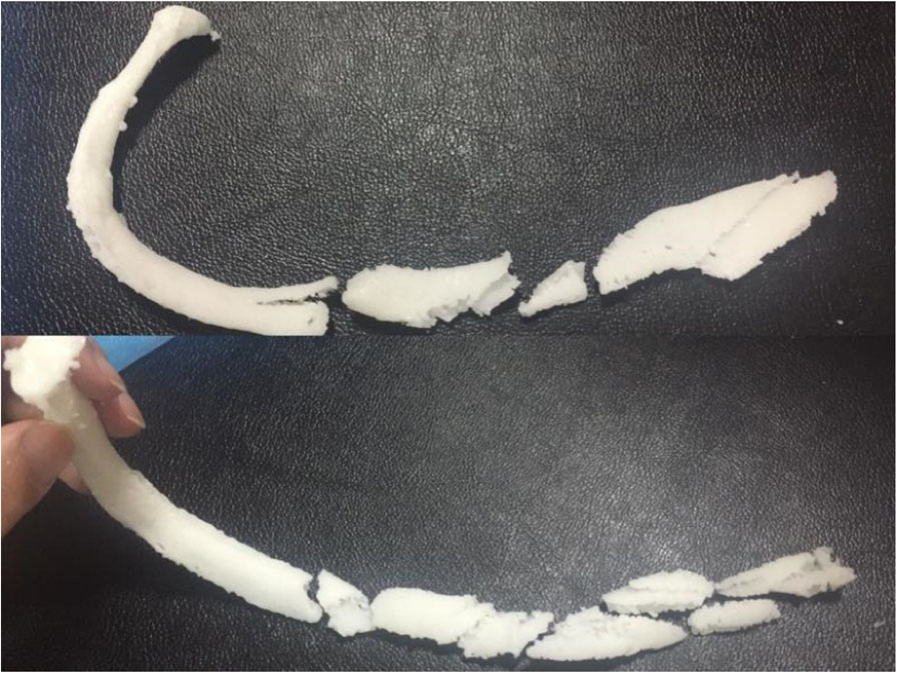
3D model printing. Restoring fracture morphology; splicing a 3D printed model

**Fig. 6:**
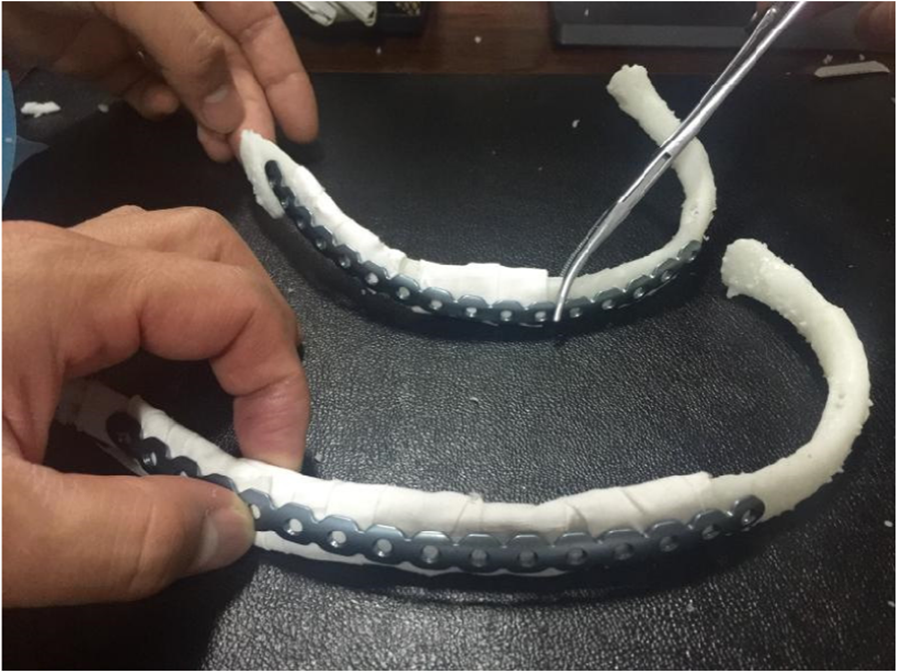
Shaping the metal plate according to the model

The two rib metal plates were separately shaped according to the reconstruction model (see Figs. 5 and 6).

**Fig. 7:**
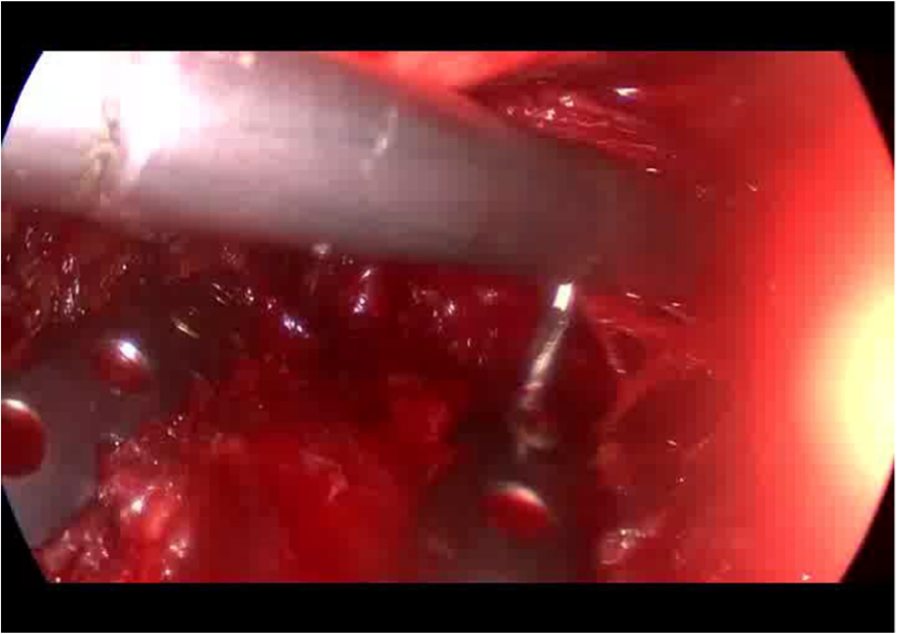
Intraoperative thoracoscopic assisted MIPO. The screws were drilled by the Intraoperative thoracoscopic assisted MIPO technique

**Fig. 8:**
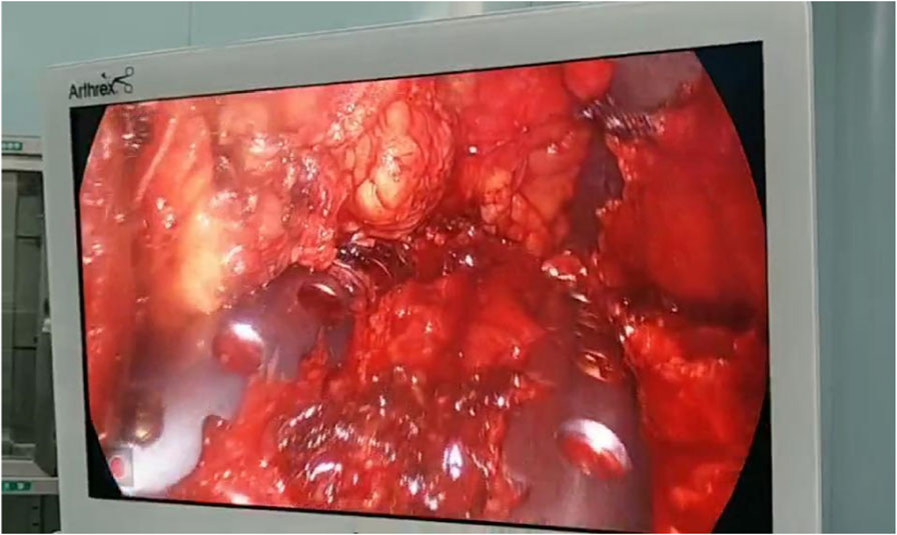
thoracoscopic observation of the ribs. The shape of the 3rd and 4th rib bone plates after being fixed

**Fig. 9:**
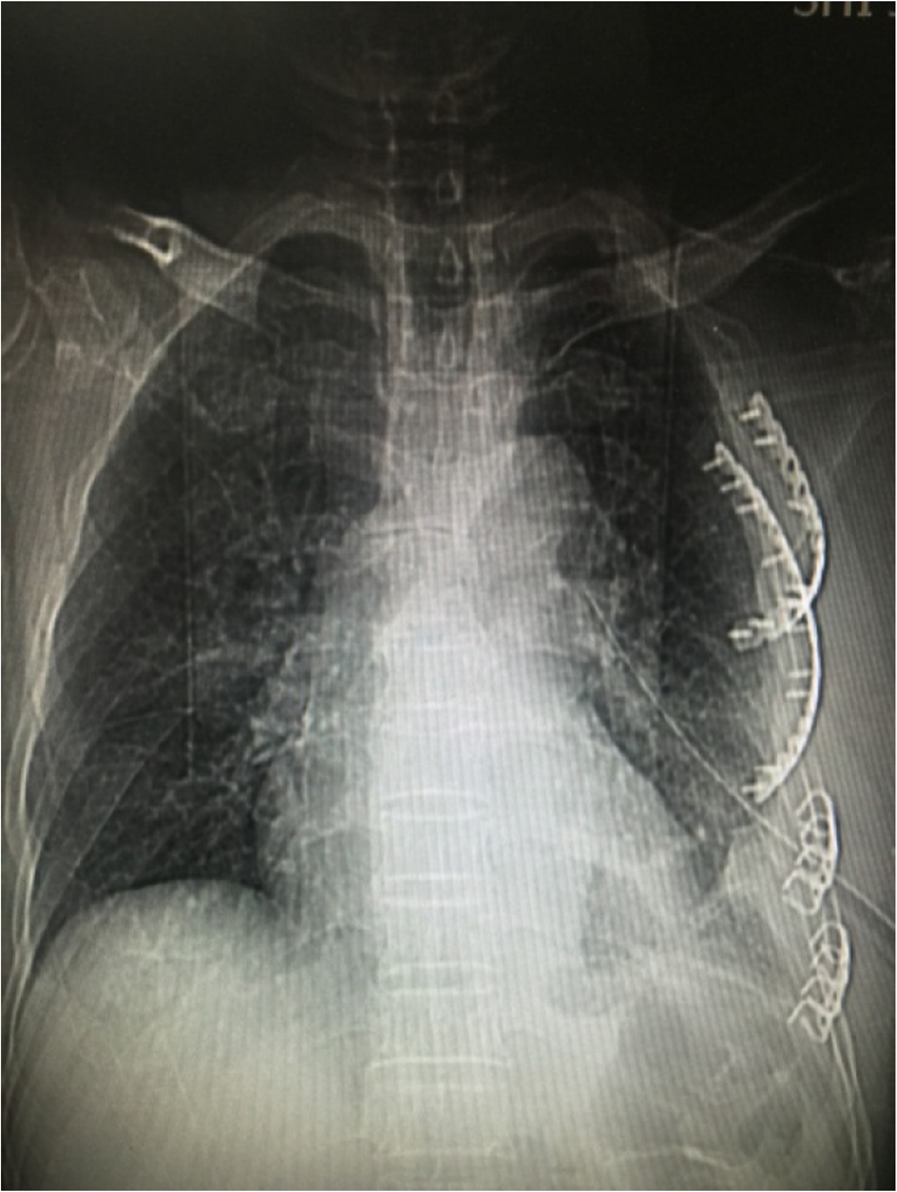
post-operation review of the chest radiograph. In post-operation review of the chest radiograph, the shape of the internal fixation is intact and completely symmetrical with the contralateral ribs

The patient is scheduled to have a open reduction and internal fixation of 3–6 rib fracture. After general anesthesia, right side lying position, small incision about 8 cm was performed under the edge of 4th rib underarm. The skin was sequentially incised and the subcutaneous tissue was freed layer by layer. The front of the latissimus dorsi muscle and the anterior serratus were exposed. The tunnel was established on the 3rd and 4th rib surfaces from the back of the chest and small muscles to the back of the scapula. The special long hooks lifted the scapula and exposed the scapular operation space. With assistance of endoscope, the electrocautery is useful to expose 3 cm outside the outermost fracture lines of the 3 and 4 ribs. The locking plate was molded on the surface of the third rib before operation, and the broken end of the non-fracture at the anterior and posterior portions of the third rib was well fitted. The distance between the two ends of the metal bone plate exceeded the fracture line to 3 nail holes distance. Under the thoracoscope, the metal plate and the ribs were temporarily fixed with long-angled forceps. The MIPO system was used to drill the holes. Two screws were implanted and locked at both ends to firmly fix the metal plate. In turn, each fracture segment was reset and drilled and secured to a metal plate. The fourth rib is fixed in the same way. Intraoperative image (Figs. 7, and 8). 5, 6 rib fractures given to fix the ribs, not the content of this article, not elaborated. Sufficient to stop the bleeding, the wound was given to leave a negative pressure drainage tube. After a routine thoracoscopic probe of the chest cavity, a closed thoracic drainage tube was placed posterior to the 7th intercostal space and the incision was closed layer by layer. After the chest wall is well-shaped. Three days after surgery review the map (Fig. 9).

## Discussion and conclusions

Rib fractures are most common in chest injuries, accounting for 40 to 60% thoracic injuries [4]. Surgical treatment of complex rib fractures has become a routine method. However, for long segment, superior, and comminuted rib fractures, due to the complex anatomical location, conventional surgical operations are more difficult and patients have greater surgical injury.

**Fig. 10:**
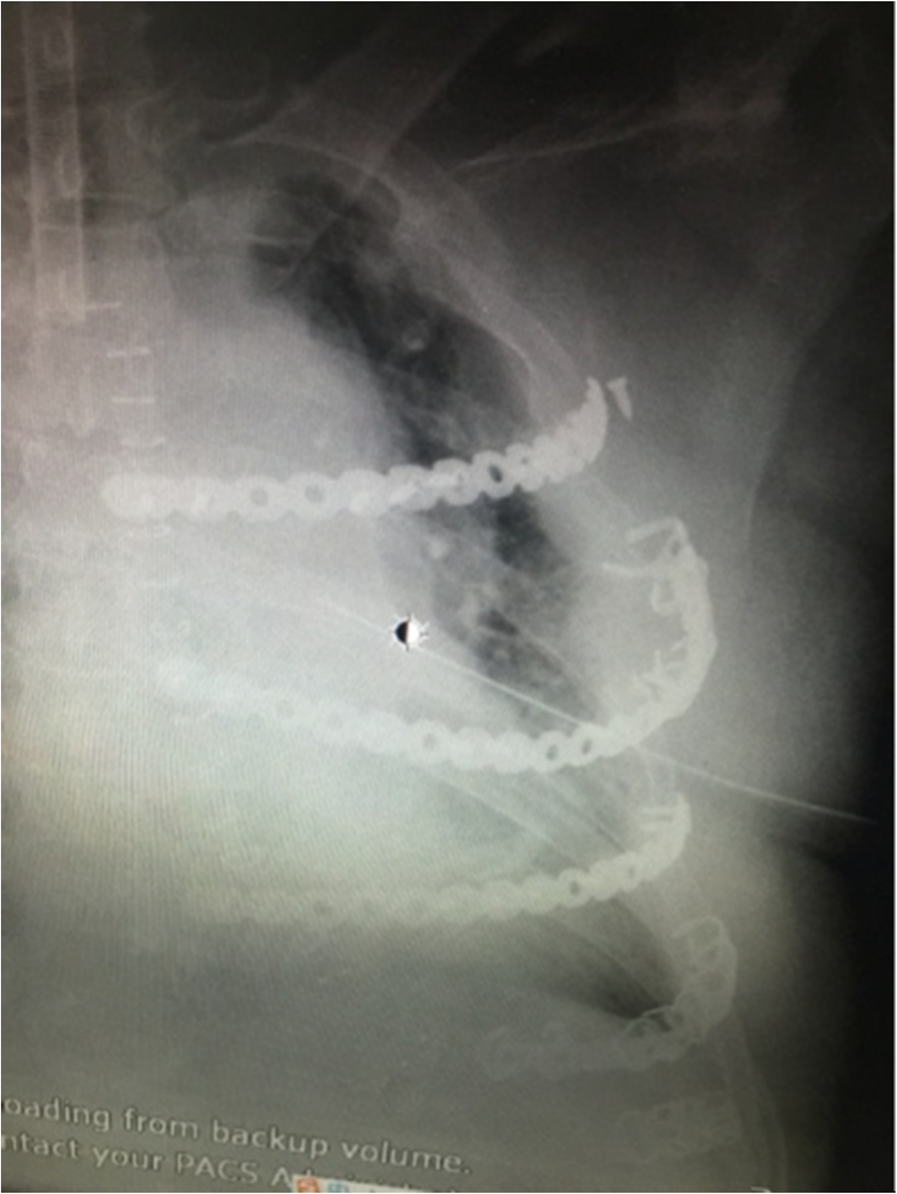
visualization of the fixed ribs. In another patient, the detachment screws are visible at the back of the uppermost metal plate. The second metal plate is not well-shaped. The posterior and ribs are poorly applied. In order to prevent falling off, it is fixed by the embracing fixator

The thorax is a complex three-dimensional structure, and the ribs also have relatively special anatomical characteristics. In addition to the conventional anatomical parameters such as length and width, bending angle and longitudinal twisting angle are also important parameters. Although the commonly used fixtures have been greatly improved, it is still difficult to fully comply with the normal physiological and anatomical characteristics of three-dimensional bending and twisting of the ribs. For long segments and crushed rib fractures, the ribs have been severely deformed, and the basis for the shaping of the fixed material has been lost, causing great difficulties for surgery, and repeated adjustment of fixed materials increases the operation time and trauma. In particular, if the poor shape of the internal fixation is poor and the two ends of the fracture are poorly attached, the internal fixation body will have a distorted tension, and the ribs are weak and continuous moving. All these will easily lead to unscrewing and fixation (see Fig. 10).

The 3D printing technology has already brought about big improvements in various fields of medicine, especially orthopaedics [5–7] and also thoracic surgery [8–11]. This study used 3D printing technology, preoperative CT thin-layer scanning, reconstruction of 3D models based on scan results, and preparation of rib models using 3D printing techniques. The fractures were modeled before the reconstruction of the normal rib morphology. Reshape the ribbed metal plate according to the reconstruction model. During the operation, the shaped metal plate is fixed on the ribs outside the fracture line to establish the basic frame, and then the other fracture segments are respectively reset and fixed on the metal plate, which saves time and labor, and is satisfactory in shaping.

Especially for long upper rib fractures, due to the anatomical relationship between scapula and other anatomies, traditional surgical methods are difficult and traumatic [12]. With the help of thoracoscopy, only a small space is needed to perfectly reveal the area under the scapula, and the fractured end is well exposed. It is easier to complete the rib drilling fixation with the MIPO system [2], especially in combination with the above-mentioned 3D printing technology for pre-shaping the steel plate. And individualized treatments provide new methods.

In summary, the combination of 3D printing technology and MIPO technology in the minimally invasive treatment of long comminuted rib fractures assisted by thoracoscopy, greatly reduced the time and improved the accuracy of intraoperative fixed plastics, reducing the difficulty of surgery and patient injury. The method still needs more clinical experience to provide better services for the majority of patients.

## Abbreviations

MIPO: Minimally Invasive Plate Oseoynthesis
